# Significant benefits of new communication technology for time delay management in STEMI patients

**DOI:** 10.1371/journal.pone.0205832

**Published:** 2018-11-02

**Authors:** Martin Studencan, Daniel Alusik, Lukas Plachy, Lubica Bajerovska, Michal Ilavsky, Jozef Karas, Adriana Kilianova, Juraj Sykora, Vladimir Hosa, Jan Kmec, Miroslav Slanina, Daniela Boguska

**Affiliations:** 1 Cardiocenter, Cardiology Clinic of the Teaching Hospital of J.A. Reiman and Prešov University, Faculty of Health Care, Prešov, Slovakia; 2 EMS Košice, Košice, Slovakia; 3 EMS Falck, Košice, Slovakia; 4 Prešov University, Faculty of Health Care, Prešov, Slovakia; University of Tampere, FINLAND

## Abstract

**Background:**

In the acute phase of STEMI, the length of the total ischemic interval is the principal factor affecting both short- and long-term mortality. The length of the interval remains a global problem, and in EU countries these figures vary between 160 and 325 min.

**Methods and results:**

The aim of our research was to assess the benefit of the systematic implementation of the new smartphone-based communication technology “STEMI” enabling immediate ECG picture and voice consultation between an EMS crew in the field and a cardiologist in the PCI-center. The transfer of ECG was associated with 92% technical success. 5 Monthly data from 2016 were compared from the reference2 monthly data set in 2015 when the data in the same area was collected in the SLOVAKS registry. The 5-months data from 2016 were compared to the reference group from 2015, when similar 2-months data in the same area in SLOVAKS registry was collected but communication technology “STEMI” technology was not used. In the monitored period in 2016 we recorded a significant decrease in unwanted secondary STEMI transportations (34.32% vs. 12.9%, p<0.001) and a significant reduction in the total ischemic interval (241 min vs. 181 min, p = 0.03). There was no significant decrease in the subinterval of “admission-pPCI” (28min vs. 23 min, p = 0.144).

**Conclusion:**

The systematic use of smartphone-based communication technology ”STEMI” enabling remote ECG picture consultation between an EMS crew and a cardiologist in PCI-center had a positive impact on the quality of care for patients with acute STEMI and brought clinical practice closer to the current ESC Guidelines. It significantly decreased the ratio of unwanted secondary transportations and led to a significant reduction in the total ischemic interval.

## Introduction

In the acute phase of STEMI, the length of the total ischemic interval is the principal factor affecting both short- and long-term mortality, but also the incidence of complications and the quality of future patient life. The optimization of the organizational measures minimizes the time delay and helps reduce the risk of death in the first few hours of STEMI and decreases the amount of myocardial damage. Preserved systolic LV function is one of the strongest favorable prognostic factors. The American and European STEMI Management Guidelines highlight the aspects of time management and minimization of the total ischemic interval [1,2]. An important component of this interval is the period from the onset of STEMI symptoms until first medical contact, for which the patient is mainly responsible for himself. This period has probably the biggest potential for reducing the time delay, but addressing this issue is more of a social than a health issue and is not the subject of this publication. As far as healthcare activities are concerned, in the past organizational measures were focused mainly on the interval from the patient’s admission to the PCI-center to the primary PCI (the “door to balloon,” or “D2B” interval). Over the past years these efforts have led to significant improvements in intrainstitutional management and reducing time delays in many countries. However, further possibilities for improving the patient prognosis by decreasing the D2B interval are already limited, as even more studies have failed to confirm the clinical benefit of this [3]. The current European and American Guidelines emphasize shifting the focus to the interval from ECG diagnosis to patient admission in the PCI-center (“ECG-door” interval), as it is possible to identify larger reserves in time management during this period. The main problem is the limited ability of the EMS crew to set the correct ECG diagnosis of STEMI on-site and the unnecessary transfers of patients to local hospitals. Once the ECG diagnosis of STEMI is established, these patients undergo secondary transport to the nearest PCI-center. This practice causes an unacceptable time delay. According to some studies, the median of the time delay due to undesirable secondary transportation is at least 60 minutes [4]. Secondary transport is also often carried out by another EMS crew. This unwanted practice is widespread worldwide and the share of secondary STEMI transportation could be 20–80%.

## Aim and methods

The aim of our research was to assess the benefit of the systematic implementation of the new communication technology “STEMI” in all EMS vehicles in the area surrounding one PCI-center. The impact on the time management of patients with acute STEMI treated with primary PCI has been taken as the focus.

**Fig 1:**
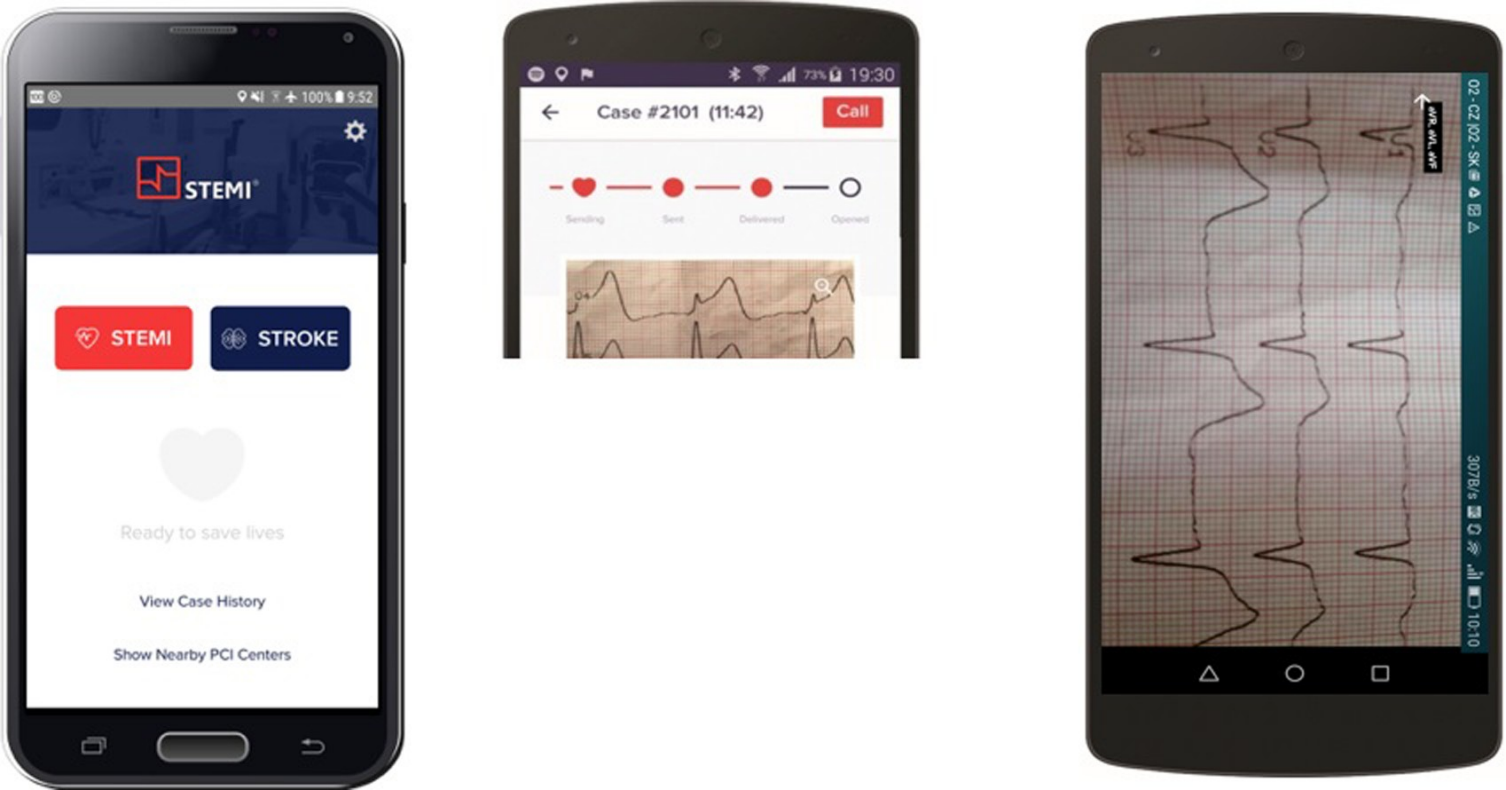
The Communication technology STEMI enabling instant ECG image and voice consultation between the EMS crew and a cardiologist in a PCI-center. (Reprinted from STEMI Global s.r.o. under a CC BY license, with permission from STEMI Global s.r.o., original copyright 2016.) ^S1 A^

The Communications Technology “STEMI” (STEMI Global Ltd.) uses the mobile internet and multi-version of a mobile application. Smartphones in every EMS vehicle and in the PCI-center (“hot line”) in a 24/7 regime have been established. The smartphone holder on the PCI-center side was a cardiologist skilled in the ECG diagnosis of STEMI and bundle branch blocks. The communication technology “STEMI” allows paramedics an instantaneous ECG picture and voice consultation of the STEMI with a cardiologist. EMS crews were required to use “STEMI” technology for remote ECG consultation only in cases of diagnostic uncertainty. Once the ECG criterion of STEMI was confirmed by the cardiologist, the PCI-center has activated the invasive team with the intention of being ready at the time of the patient’s arrival at the cathlab. In cases of confirmed STEMI diagnosis, the EMS crew was always required to perform the patient’s transport directly to the cathlab. Additional features of the communication technology “STEMI” include GPS utilization and estimated time of arrival calculation (ETA), automatic estimation of the ECG-pPCI interval, and warning when time criteria justify thrombolysis (“TA-thrombolysis alert” function). The cardiologist’s smartphone enables the monitoring of EMS vehicle position on the map and ETA, which makes it possible to adapt the preparation of the cathlab to the current information (Fig 1).

We analyzed a period of 5 months 8-12/2016, when “STEMI” technology was applied to all, i.e. 52 EMS vehicles, in the area of Eastern Slovakia surrounding the PCI- center of the Teaching Hospital of J.A. Reiman in Prešov. The observed data, along with the demographic characteristics, included the length of the total ischemic interval, the time of ECG diagnosis, the time of the patient’s admission in the PCI-center, the time of primary PCI, and information about the primary/secondary form of transportation. The data obtained were compared with similar data from 2015 when a 2-month cross-sectional survey of the SLOVAKS registry was undertaken in Slovakia, including the PCI-center Prešov [5]. In the reference period in 2015, neither communication technology “STEMI” nor additional technology enabling remote ECG consultation with a PCI-center has been used. In both compared periods the data collection was conducted in the same way. The time of onset of the symptoms was determined according to the clinical judgment of the cardiologist, and for the ECG diagnosis the time data printed on the first diagnostic ECG record was decisive. The patient’s admission time was considered to be the time of taking over the patient from the lounger of the EMS crew to the bed of the PCI-center or cathlab table. The PCI time as recommended by ESC Guidelines was considered the time of crossing the culprit lesion by PCI wire.

### Statistics

Statistical analysis was performed using the SPSS program. Descriptive statistics were used for the description of the sample. χ^2^-tests were used to explore associations between the nominal variables and Mann-Whitney U-tests for the analysis of total ischemic period changes.

## Results

In the monitored 5-month period in 2016, EMS crews carried out 184 ECG consultations using the Communication technology “STEMI.” Ultimately, the STEMI diagnosis was confirmed and the primary PCI performed on 50 patients from 184 consulted cases. The decision to use or not to use “STEMI” technology in individual cases for remote ECG consultation was up to EMS staff. It copy the official recommendation to use “STEMI” technology only in cases of diagnostic uncertainty. The decision to transfer the patient directly to the PCI-center was based on the cardiologist’s opinion. There were next 128 confirmed STEMI patients admitted to the PCI-center using different ways of transportation (i.e. selftransported) or EMS help with no use of “STEMI“technology. So all together 178 patients, 43,5% females, with a confirmed diagnosis of acute STEMI were hospitalized in the PCI-center. For all these patients primary PCI was performed. The interval “symptoms-ECG“〈 120 min reached 64% of them. As to the age and the ejection fraction, there was not any significant difference between the analyzed group from 2016 (n = 155) and the reference group from 2015 (n = 67) (Table 1).

**Table 1:** Comparison of the clinical characteristics between the analyzed group (2016) and the reference group (2015).

	year		
	**2015**	**2016**	
**period**	2 mes.	5 mes.	
**n**	67	178	
**"symptoms-ECG" interval 〈 120 min**	53%	64%	
**female**	52%	43,50%	
**age**	61,66	64,66	p = 0,105
**EF(%)**	46,11	43,45	p = 0,065

**Fig 2:**
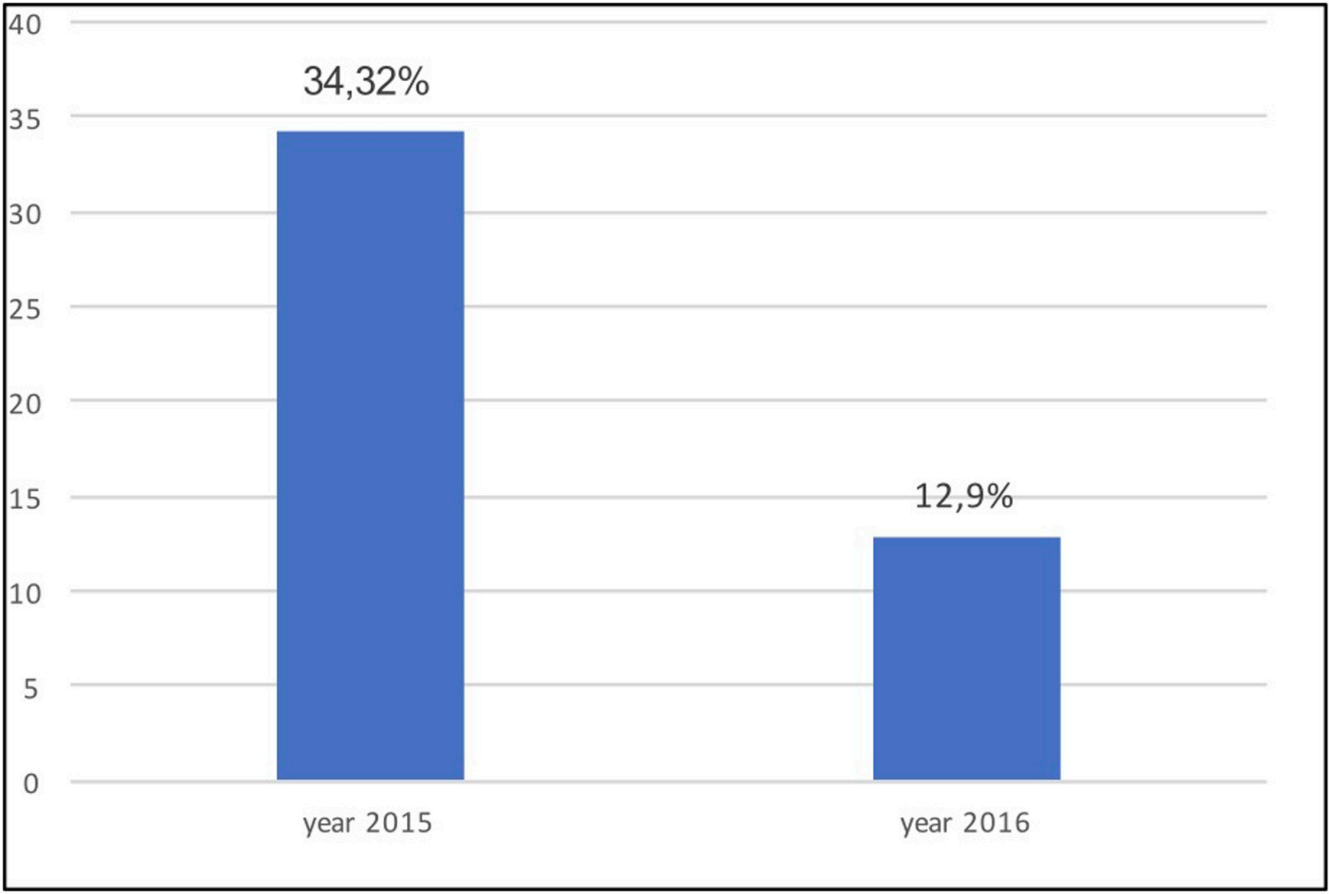
The comparison of the share of secondary transportations between the reference group from 2015 and the analyzed group from 2016 when STEMI communication technology was used (p<0.001).^S2 A^

**Fig 3:**
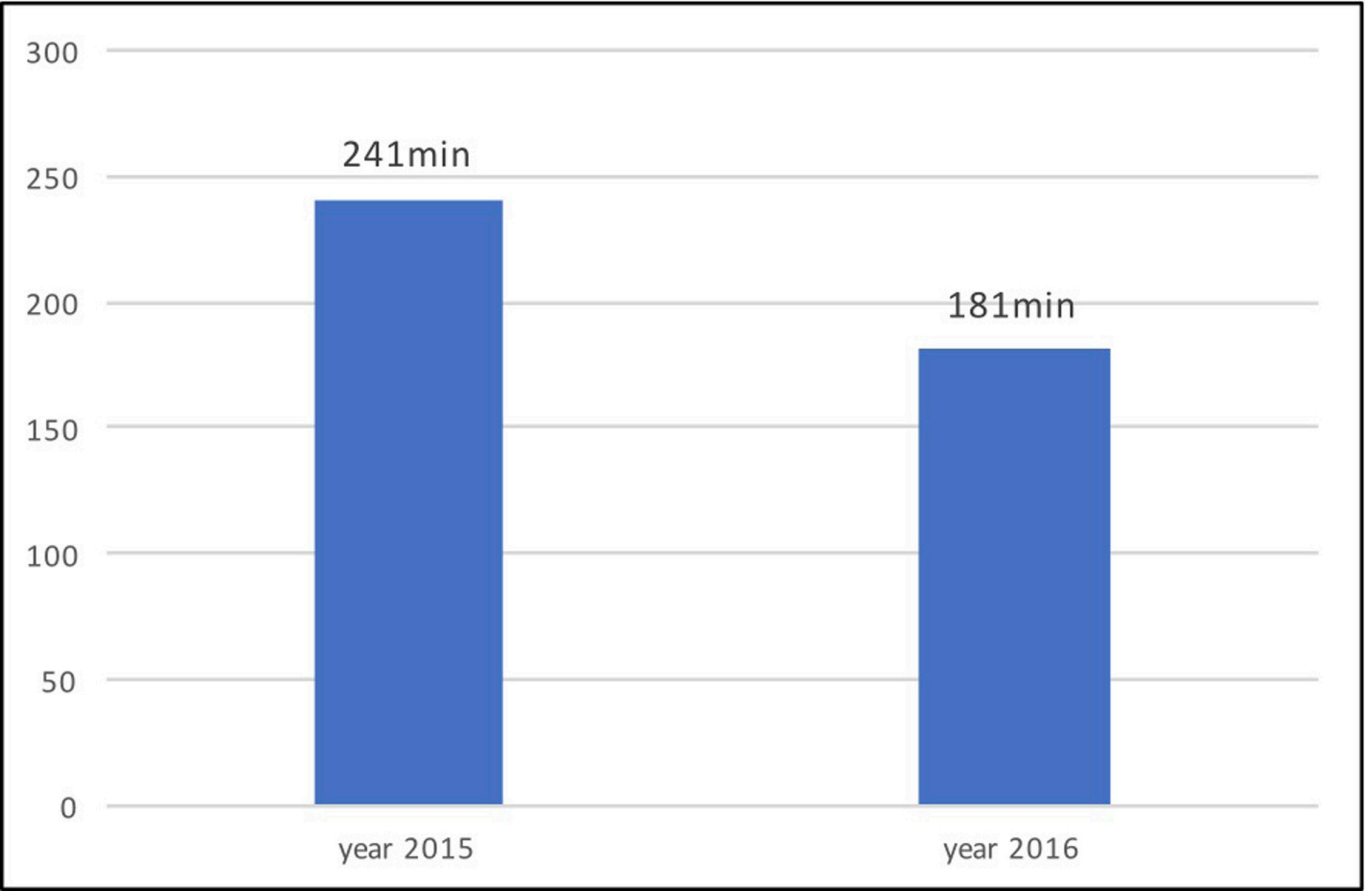
The comparison of the medians of the total ischemic interval between the reference group from 2015 and the analyzed group from 2016 when STEMI communication technology was used (p = 0.03).^S1 S^

In the reference group from 2015, the share of undesirable secondary transportations was 34.32%, while in the analyzed group from 2016 it decreased to 12.9% with the use of STEMI communication technology (p<0.001). The comparison of the share of secondary transports between the groups is shown in Fig 2. The median of the total ischemic interval in the reference group was 241(±257) minutes, dropping to 181(±172) minutes in the analyzed group from 2016 (p = 0.03) (Fig 3).

According to the current ESC Guidelines, primary PCI should be performed not later than 120 minutes from the ECG diagnosis, but ideally within 90 minutes of ECG diagnosis. In the reference group, the criterion “within 120 minutes” was met by 66% of patients and the optimal criterion “within 90 minutes” by just 25% of patients. In the analyzed group, the proportion of patients “within 120 minutes” increased to 71% (p = 0.508) and “within 90 minutes” reached 35% (p = 0.184), thus the share increments were not significant.

**Fig 4:**
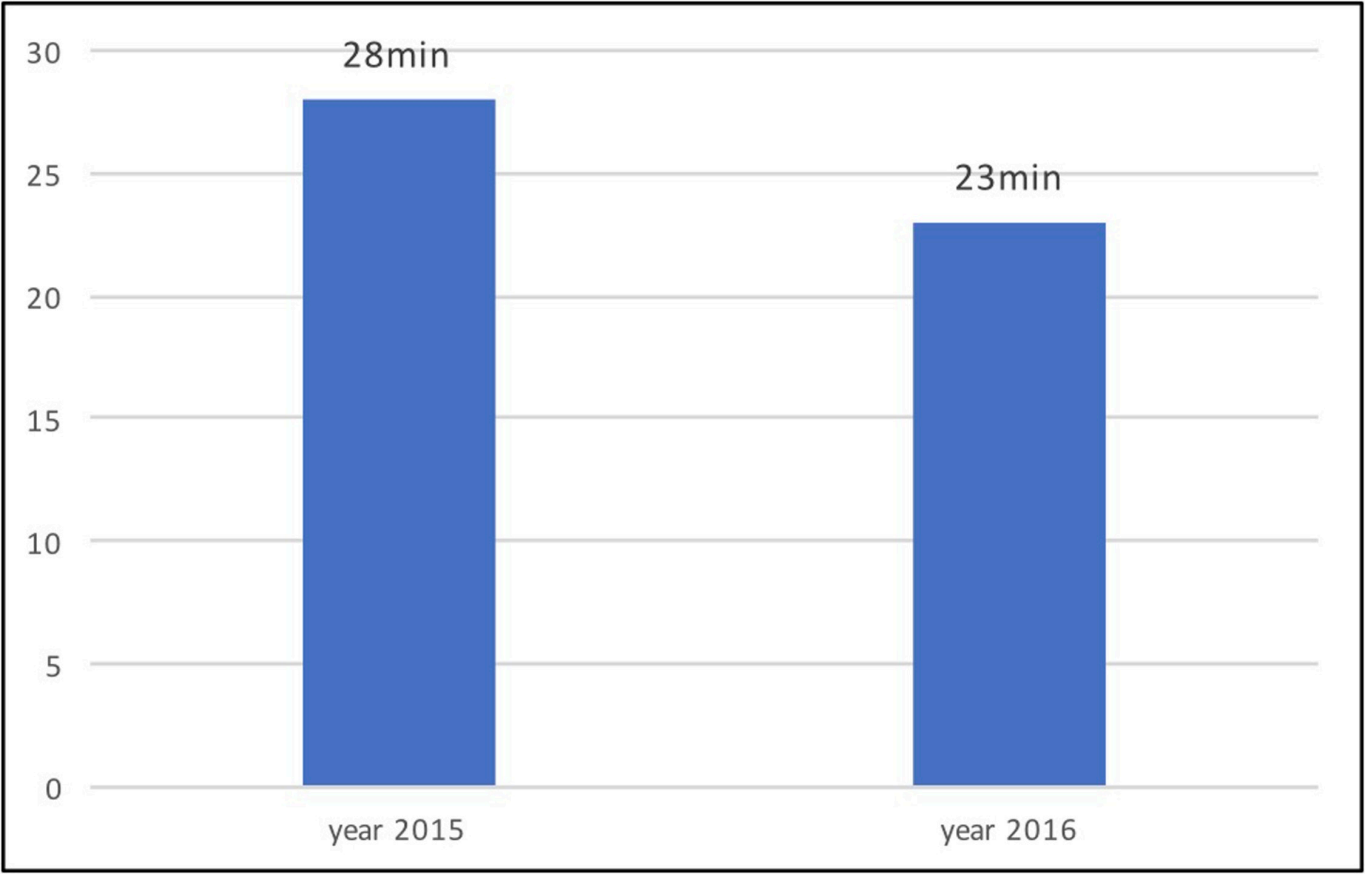
The comparison of the medians of the “admission-pPCI” interval between the reference group from 2015 and the analyzed group from 2016 when using STEMI communication technology (p = 0.144).^S2 S^

In the 2015 reference group, the median of the “admission-pPCI” interval was 28(±16) minutes, and in the analyzed group from 2016 it was 23(±13.1) minutes. This difference did not reach statistical significance (p = 0.144) (Fig 4).

## Discussion

**Fig 5:**
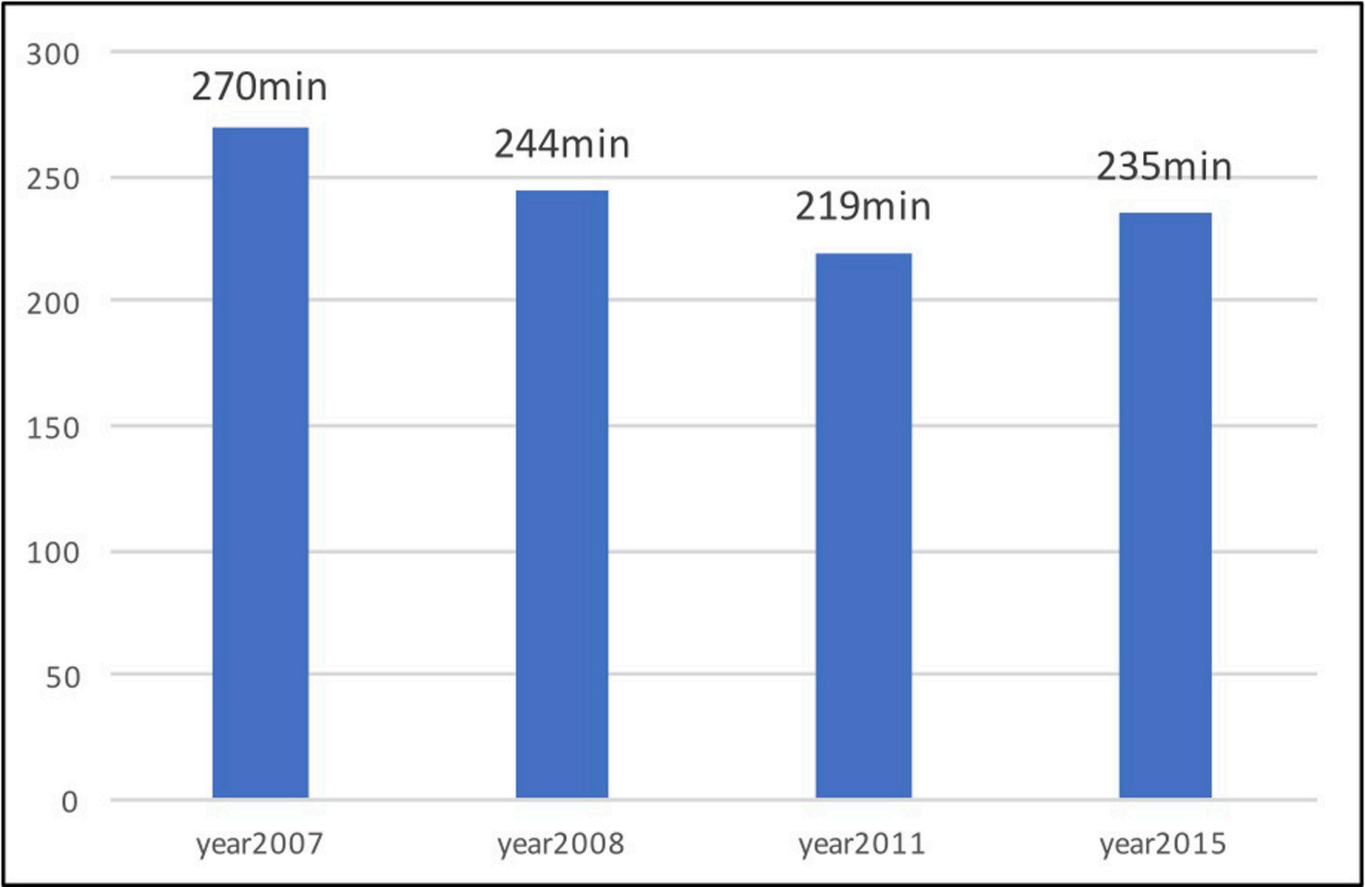
The total ischemic time for patients with STEMI treated with pPCI in Slovakia according to the results of the national registry of Acute Coronary Syndromes SLOVAKS [5].^S3 S^

The length of the total ischemic interval in patients with STEMI is a problem in many countries. According to the published data, in EU countries these figures vary between 160 and 325 min [6]. In Slovakia, following the methodological guidance of the Ministry of Health [7], the median of this interval has decreased from 270 to 244 minutes in 2008. But over the last 10 years, we have not been successful in decreasing it further, and it still reaches up to 4 hours (Fig 5).

The systematic implementation of the communication technology “STEMI” and the improvement of time management of STEMI patients in the defined geographic area of Eastern Slovakia managed to reduce the total ischemic interval in a short time from 241 to 181 minutes, which is already among the levels of the best EU countries. The fact that the length of the overall ischemic interval is closely correlated with the patient’s prognosis as to the survival and quality of life is generally known. This work documented the fact that the emphasis on organizational measures and improvement of time management can fundamentally influence the prognosis of STEMI patients, and the investment in these financially low-cost measures is surprisingly highly effective.

According to the study results, using of “STEMI” technology in 2016 did not decrease significantly “ECG-pPCI” interval. This finding needs an explanation. A significant increase in the proportion of patients whose pPCI was performed within the time limit was not mathematically expected, which was related to the methodology. For “ECG time,” the time when the ECG was evaluated as STEMI was considered. In 2015, in many cases the EMS crew in the field recorded an ECG, but subsequently the patient was transferred to a local hospital for STEMI diagnosis where a new ECG was made and was STEMI diagnosed afterwards. For the statistical need, ECG time was used from a local hospital. In 2016, EMS crews in the field recorded an ECG and many times using “STEMI” technology immediately consulted with a cardiologist, so the primary transportation was intended. In this case, “ECG time” in the field was a matter of course used for the statistics. It is clear that in similar situations in 2015 and 2016, the recorded ECG-pPCI intervals could be approximately the same, but in 2016, due to primary transportation, there was certainly a reduction in the total ischemic interval. Thus, from 2015 and 2016, methodologically different “ECG-pPCI” intervals were compared.

Remote ECG consultation and a quicker activation of the invasive team provide the theoretical prerequisites for improving the interinstitutional measures and decreasing the “admission-pPCI” interval. In this study, the “admission-pPCI” was shortened from 28 to 23 minutes, but this was not a significant difference (p = 0.144). The analyses of the SLOVAKS registry [5] at our workplace and in general in Slovakia over the last several years have been documented at less than 30 minutes for the “admission-pPCI” interval, which is a time well above standard and its significant reduction is probably not further possible. A significant influence on this interval by the use of communication technology can be expected in workplaces where the “admission-pPCI” is still longer, mostly due to the delayed activation of the invasive team once the patient has arrived to the PCI-center, or due to unnecessary transient patient stops at the emergency room or coronary care unit.

The development of intervention techniques and increased availability of PCI-centers have brought euphoria and enthusiasm for the invasive primary PCI procedures, but the clinicians, health care insurance companies, and official authorities silently ignore the fact that a large proportion of primary PCIs are performed outside the recommended time limit, which may mean the patient’s iatrogenization. In order to guarantee evidence-based medicine, it is necessary to more often indicate pre-hospital thrombolysis and the so-called pharmacoinvasive strategy, or to optimize time management so that primary reperfusion therapy via pPCI is performed as soon as possible but definitely within a given time limit.

The unwanted secondary transportations of patients with STEMI have a remarkable effect on the length of the total ischemic interval and remain a serious medical problem with a negative impact on the prognosis of STEMI patients. This is a universal problem and studies in recent years have shown that even in countries with a well-developed health care system, the share of secondary STEMI transports is 39–50% [8–11].

Ideally, every paramedic and rescuer should be able to analyze the ECG in the field with the same reliability as the cardiologist on the CCU. In practice, however, it is not and probably will not be possible. This is related to the relatively frequent exchange of staff in the EMS segment, but also the fact that the EMS crew even after proper theoretical training need to consolidate their ECG knowledge in long-term clinical practice. However, our analysis shows that while in PCI-centers the analysis of ECG with a suspicion of STEMI is a daily routine, it is a relatively rare occurrence (one in 1–2 months) from the point of view of a particular individual paramedic. However, this must not change the ambition of the 12-lead ECG, and the focus on the ST-elevation and bundle branch blocks must be an important part of the pregraduate and postgraduate training for paramedics. In addition to the documented clinical significance of remote ECG consultation, there is one more unique dimension of the communication technology “STEMI”–educational. EMS crews are given the opportunity to immediately and repeatedly confront their personal opinions on the ECG curve with the opinion of a cardiologist-specialist.

For many years, modern ECG devices have enabled automated computer-based analysis of the ECG curve, including ST-elevation and bundle branch blocks, which could also be applicable to the prehospital management of STEMI patients. However, this approach involves certain risks. In STEMI, several papers have documented the high specificity, but limited sensitivity, of computer diagnostics. In addition, the ECG finding must always be confronted with the nature of the patient’s difficulties. Another option is the classic ECG-telemetry, which nevertheless has never reached the level of general use despite its 40 years of existence. Its main limitation was the 11–20% frequency of technical failures in the past [12], long ECG transmission time (2–4 minutes), ECG transmission to fixed PC stations or faxes, high costs of the hardware and software equipment, and also the limitation of simple ECG picture information. The communication technology “STEMI”, compare to classic “ECG-telemetry” is cheaper much more and payment is based on 1-year licence. It uses modern technological features that give it a new dimension. The ECG image is received by the cardiologist directly on the handheld device–smartphone, which allows for an immediate response. There are more functions which go far above simple ECG picture transmission. It uses GPS and enables automatic time of arrival calculation, ECG-pPCI interval estimation, graphical warning of thrombolysis indication, and so on. It enables the cardiologist to monitor the position of the ambulance on the map and a practical is overview of case histories, and database outputs. The developmental flexibility of the technology is unlimited, and the STROKE module for the early management of stroke patients is now available too (Fig 1).

The limitations of “STEMI” technology are related to the need for mobile internet access. Where coverage is lacking, the EMS crews have to work the “old-fashioned” way. There is about 70–98% coverage for mobile internet in EU countries, and large mobile operators are constantly improving this access. To date, the numbers already mentioned are unlikely to be true anymore.

## Conclusion

In patients with acute myocardial infarction with ST elevation on their ECG (STEMI), the reduction in total ischemic interval plays a crucial role in influencing the prognosis. Health care professionals are responsible mainly for the interval from the first medical contact to reperfusion. Our analysis and international experience show remarkable shortcomings in the prehospital time management of STEMI patients, which leads to the fact that a large proportion of patients treated by primary PCI pass the procedure outside of the recommended time limit and patients eligible for thrombolysis or pharmacoinvasive strategy are not receiving it.

In this study, we have analyzed the impact of the use of the communication technology “STEMI”, which enables immediate ECG picture and voice consultation between paramedics and a cardiologist in a PCI-center for the prehospital management of STEMI patients. The transfer of ECG was associated with 92% technical success. In the monitored period we recorded a significant decrease in unwanted secondary STEMI transportations and a significant reduction in the total ischemic interval.

The introduction of new communication technology into the regional PCI-center and all surrounding EMS vehicles has had a significant impact on the quality of care for patients with acute STEMI and has brought clinical practice closer to the current ESC Guidelines.

## Supporting information

S1 Appendix Publication permission. (PDF)

S2 Appendix Ethics committee statement. (PDF)

S1 Supporting information Dataset–statistic calculation. (DOCX)

S2 Supporting information Dataset- basic dataset. (XLSX)

S3 Supporting information Dataset–data for statistic calculation. (XLSX)
