# Supplementary material for: Significant benefits of new communication technology for time delay management in STEMI patients
Source: PLoS One. 2018 Nov 2;13(11):e0205832. doi: 10.1371/journal.pone.0205832 (PMC6214513; doi:10.1371/journal.pone.0205832)
Supplement: S1 Appendix — (PDF) [file pone.0205832.s001.pdf]

STEMI® STEMI Global, s.r.o., Saving Minutes, Adding Life

3rd Sep 2018

To: Editor's Office PLOSE ONE

Hereby I confirm that STEMI Global s.r.o. is Copyright holder of STEMI technology since 2016. The technology is based on telemedicine principles and mobile application. It enables immediate picture and voice consultation between paramedics on field and specialists in hospitals.

I give the permission for the open-access journal PLOS ONE to publish screens of mobile application STEMI under the Creative Commons Attribution License (CCAL) CC BY 4.0.

Yours Faithfully,

Milan Barger, CEO of STEMI Global s.r.o.

Kontakt:
Web: stemiglobal.sk (com)
Email: info@stemiglobal.com

Bankové spojenie:
IBAN: SK11 1100 0000 0029 4303 4723
SWIFT: TATRSKBX

IČO: 50710541
DIČ: 2120426330
IČ DPH: SK2120426330
