# Supplementary material for: Significant benefits of new communication technology for time delay management in STEMI patients
Source: PLoS One. 2018 Nov 2;13(11):e0205832. doi: 10.1371/journal.pone.0205832 (PMC6214513; doi:10.1371/journal.pone.0205832)
Supplement: S2 Appendix — (PDF) [file pone.0205832.s002.pdf]

FAKULTNÁ NEMOCNICA S POLIKLINIKOU

J. A. REIMANA PREŠOV

doc. MUDr. Martin Studenčan, PhD.
Kardiocentrum FNsP J. A. Reimana Prešov

Vec: Statement

Statement on the planned retrospective observational study, which will retrospectively evaluate the use of new communication technology STEMI based on telemedicine. The technology enabled remote voice and screen (ECG) consultation between field rescuers (EMS) and a cardiology specialist. Technology has accelerated the timely management of patients with myocardial infarction, and it is possible to predict improvement in prognosis in these patients.

Title: The benefit of telemedicine for early management of patients suffering from myocardial infarction.
Workingplace: Cardiocentre of FNsP J.A.Reiman, Prešov
Principle investigator: assoc.prof. MUDr.Martin Studenčan. M.D., PhD.
Period as a subject of planned analysis: 01.08.-31.12.2016

The plan is a retrospective observational analysis of real clinical practice. The use of STEMI communication technology has not changed standard treatment. The use of this technology was left to rescuers (EMS staff) who, in case of uncertainty in the diagnosis of myocardial infarction, had the opportunity to use the technology for remote ECG consultation with a specialist. Given the nature of this analysis, informed patient consent was not needed, and the official approval of the Ethics Committee is not needed to carry out the analysis.

V Prešove 12.1.2017

Fakultná nemocnica s poliklinikou
J. A. Reimana Prešov
ETICKÁ KOMISIA
Hollého 14, 081 81 Prešov

MUDr. Marek Paľo, PhD., MBA
Predseda Etickej komisie FNsP J. A. Reimana Prešov
