# Supplementary material for: Significant benefits of new communication technology for time delay management in STEMI patients
Source: PLoS One. 2018 Nov 2;13(11):e0205832. doi: 10.1371/journal.pone.0205832 (PMC6214513; doi:10.1371/journal.pone.0205832)
Supplement: S1 Supporting information — (DOCX) [file pone.0205832.s003.docx]

**Age T-test**

| **Group Statistics** | | | | | |
| --- | --- | --- | --- | --- | --- |
|  | rok | N | Mean | Std. Deviation | Std. Error Mean |
| vek | 2015 | 67 | 61,66 | 12,111 | 1,480 |
|  | 2016 | 178 | 64,66 | 13,143 | ,985 |

| **Independent Samples Test** | | | | | | | | | | |
| --- | --- | --- | --- | --- | --- | --- | --- | --- | --- | --- |
|  | | Levene's Test for Equality of Variances | | t-test for Equality of Means | | | | | | |
|  |  | F | Sig. | t | df | Sig. (2-tailed) | Mean Difference | Std. Error Difference | 95% Confidence Interval of the Difference | |
|  |  |  |  |  |  |  |  |  | Lower | Upper |
| vek | Equal variances assumed | ,000 | ,986 | **-1,626** | 243 | **,105** | -3,001 | 1,845 | -6,634 | ,633 |
|  | Equal variances not assumed |  |  | -1,688 | 128,097 | ,094 | -3,001 | 1,778 | -6,518 | ,517 |

**There was not singif. difference in age between 2015 and 2016**

**Primary transport χ2-test**

**Crosstabs**

| **Notes** | | |
| --- | --- | --- |
| Output Created | | 08-JUL-2017 23:17:03 |
| Comments | |  |
| Input | Active Dataset | DataSet0 |
|  | Filter | <none> |
|  | Weight | <none> |
|  | Split File | <none> |
|  | N of Rows in Working Data File | 245 |
| Missing Value Handling | Definition of Missing | User-defined missing values are treated as missing. |
|  | Cases Used | Statistics for each table are based on all the cases with valid data in the specified range(s) for all variables in each table. |
| Syntax | | CROSSTABS  /TABLES=rok BY primarnytransport  /FORMAT=AVALUE TABLES  /STATISTICS=CHISQ  /CELLS=COUNT  /COUNT ROUND CELL. |
| Resources | Processor Time | 00:00:00,03 |
|  | Elapsed Time | 00:00:00,06 |
|  | Dimensions Requested | 2 |
|  | Cells Available | 524245 |

| **Case Processing Summary** | | | | | | |
| --- | --- | --- | --- | --- | --- | --- |
|  | Cases | | | | | |
|  | Valid | | Missing | | Total | |
|  | N | Percent | N | Percent | N | Percent |
| rok * primárny transport | 222 | 90,6% | 23 | 9,4% | 245 | 100,0% |

| **year * primary transport Crosstabulation** | | | | |
| --- | --- | --- | --- | --- |
| Count | | | | |
|  | | primary transport | | Total |
|  |  | no | yes |  |
| year | 2015 | 23 | 44 | 67 |
|  | 2016 | 20 | 135 | 155 |
| Total | | 43 | 179 | 222 |

| **Chi-Square Tests** | | | | | |
| --- | --- | --- | --- | --- | --- |
|  | Value | df | Asymptotic Significance (2-sided) | Exact Sig. (2-sided) | Exact Sig. (1-sided) |
| Pearson Chi-Square | **13,749^a^** | **1** | **,000** |  |  |
| Continuity Correction^b^ | 12,412 | 1 | ,000 |  |  |
| Likelihood Ratio | 12,846 | 1 | ,000 |  |  |
| Fisher's Exact Test |  |  |  | ,000 | ,000 |
| Linear-by-Linear Association | 13,688 | 1 | ,000 |  |  |
| N of Valid Cases | 222 |  |  |  |  |
| a. 0 cells (0,0%) have expected count less than 5. The minimum expected count is 12,98. | | | | | |
| b. Computed only for a 2x2 table | | | | | |

**The primary transportation in 2016 was signiff. higher compare to that one in 2015.**

**Total ischemic interval Mann-Whitney U-test**

| **Descriptives** | | | | | |
| --- | --- | --- | --- | --- | --- |
|  | year | | | Statistic | Std. Error |
| Total ischemic interval | 2015 | Mean | | 322,56 | 34,074 |
|  |  | 95% Confidence Interval for Mean | Lower Bound | 254,30 |  |
|  |  |  | Upper Bound | 390,82 |  |
|  |  | 5% Trimmed Mean | | 286,51 |  |
|  |  | Median | | 241,00 |  |
|  |  | Variance | | 66181,072 |  |
|  |  | Std. Deviation | | 257,257 |  |
|  |  | Minimum | | 115 |  |
|  |  | Maximum | | 1305 |  |
|  |  | Range | | 1190 |  |
|  |  | Interquartile Range | | 228 |  |
|  |  | Skewness | | 2,320 | ,316 |
|  |  | Kurtosis | | 5,521 | ,623 |
|  | 2016 | Mean | | 229,53 | 16,205 |
|  |  | 95% Confidence Interval for Mean | Lower Bound | 197,42 |  |
|  |  |  | Upper Bound | 261,64 |  |
|  |  | 5% Trimmed Mean | | 209,73 |  |
|  |  | Median | | 181,00 |  |
|  |  | Variance | | 29674,716 |  |
|  |  | Std. Deviation | | 172,264 |  |
|  |  | Minimum | | 35 |  |
|  |  | Maximum | | 1505 |  |
|  |  | Range | | 1470 |  |
|  |  | Interquartile Range | | 142 |  |
|  |  | Skewness | | 4,127 | ,227 |
|  |  | Kurtosis | | 26,528 | ,451 |


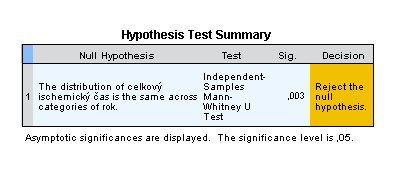


**Total ischemic interval was significantly lower in 2016 compare to thath one in 2015**

**Ejection fraction T-test**

| **Group Statistics** | | | | | |
| --- | --- | --- | --- | --- | --- |
|  | year | N | Mean | Std. Deviation | Std. Error Mean |
| EF | 2015 | 66 | 46,106 | 10,0447 | 1,2364 |
|  | 2016 | 150 | 43,447 | 9,5545 | ,7801 |

| **Independent Samples Test** | | | | | | | | | | |
| --- | --- | --- | --- | --- | --- | --- | --- | --- | --- | --- |
|  | | Levene's Test for Equality of Variances | | t-test for Equality of Means | | | | | | |
|  |  | F | Sig. | t | df | Sig. (2-tailed) | Mean Difference | Std. Error Difference | 95% Confidence Interval of the Difference | |
|  |  |  |  |  |  |  |  |  | Lower | Upper |
| EF | Equal variances assumed | ,060 | ,807 | **1,855** | 214 | **,065** | 2,6594 | 1,4337 | -,1665 | 5,4853 |
|  | Equal variances not assumed |  |  | 1,819 | 118,839 | ,071 | 2,6594 | 1,4620 | -,2355 | 5,5543 |

**There was not significant difference as to the EF between 2016 and 2015**

**EKG – PKI interval**

| **Descriptives** | | | | | |
| --- | --- | --- | --- | --- | --- |
|  | year | | | Statistic | Std. Error |
| EKG PKI | 2015 | Mean | | 112,98 | 7,228 |
|  |  | 95% Confidence Interval for Mean | Lower Bound | 98,50 |  |
|  |  |  | Upper Bound | 127,47 |  |
|  |  | 5% Trimmed Mean | | 108,43 |  |
|  |  | Median | | 111,50 |  |
|  |  | Variance | | 2925,836 |  |
|  |  | Std. Deviation | | 54,091 |  |
|  |  | Minimum | | 31 |  |
|  |  | Maximum | | 425 |  |
|  |  | Range | | 394 |  |
|  |  | Interquartile Range | | 43 |  |
|  |  | Skewness | | 3,467 | ,319 |
|  |  | Kurtosis | | 19,868 | ,628 |
|  | 2016 | Mean | | 106,76 | 3,749 |
|  |  | 95% Confidence Interval for Mean | Lower Bound | 99,34 |  |
|  |  |  | Upper Bound | 114,19 |  |
|  |  | 5% Trimmed Mean | | 104,61 |  |
|  |  | Median | | 105,00 |  |
|  |  | Variance | | 1601,881 |  |
|  |  | Std. Deviation | | 40,024 |  |
|  |  | Minimum | | 32 |  |
|  |  | Maximum | | 250 |  |
|  |  | Range | | 218 |  |
|  |  | Interquartile Range | | 47 |  |
|  |  | Skewness | | ,924 | ,226 |
|  |  | Kurtosis | | 1,677 | ,449 |

**Mann-Whitney U-test**


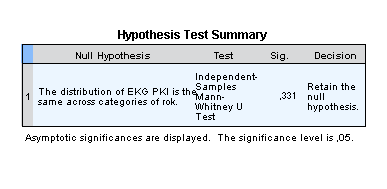


**χ^2^-test**

| **Crosstab** | | | | |
| --- | --- | --- | --- | --- |
| Count | | | | |
|  | | EKG-PKI | | Total |
|  |  | nad 120 minút | do 120 minút |  |
| rok | 2015 | 19 | 37 | 56 |
|  | 2016 | 33 | 81 | 114 |
| Total | | 52 | 118 | 170 |

| **Chi-Square Tests** | | | | | |
| --- | --- | --- | --- | --- | --- |
|  | Value | df | Asymptotic Significance (2-sided) | Exact Sig. (2-sided) | Exact Sig. (1-sided) |
| Pearson Chi-Square | **,439^a^** | 1 | **,508** |  |  |
| Continuity Correction^b^ | ,236 | 1 | ,627 |  |  |
| Likelihood Ratio | ,435 | 1 | ,510 |  |  |
| Fisher's Exact Test |  |  |  | ,596 | ,312 |
| Linear-by-Linear Association | ,436 | 1 | ,509 |  |  |
| N of Valid Cases | 170 |  |  |  |  |
| a. 0 cells (0,0%) have expected count less than 5. The minimum expected count is 17,13. | | | | | |
| b. Computed only for a 2x2 table | | | | | |

| **Crosstab** | | | | |
| --- | --- | --- | --- | --- |
| Count | | | | |
|  | | ELG-PKI | | Total |
|  |  | nad 90 minút | do 90 minút |  |
| rok | 2015 | 42 | 14 | 56 |
|  | 2016 | 74 | 40 | 114 |
| Total | | 116 | 54 | 170 |

| **Chi-Square Tests** | | | | | |
| --- | --- | --- | --- | --- | --- |
|  | Value | df | Asymptotic Significance (2-sided) | Exact Sig. (2-sided) | Exact Sig. (1-sided) |
| Pearson Chi-Square | **1,763^a^** | 1 | **,184** |  |  |
| Continuity Correction^b^ | 1,328 | 1 | ,249 |  |  |
| Likelihood Ratio | 1,805 | 1 | ,179 |  |  |
| Fisher's Exact Test |  |  |  | ,221 | ,124 |
| Linear-by-Linear Association | 1,753 | 1 | ,186 |  |  |
| N of Valid Cases | 170 |  |  |  |  |
| a. 0 cells (0,0%) have expected count less than 5. The minimum expected count is 17,79. | | | | | |
| b. Computed only for a 2x2 table | | | | | |

**There was not signifficant difference in median of EKG-PKI interval between 2016 and 2015**
